# Supplementary material for: Dysregulated inflammation in solid tumor malignancy patients shapes polyfunctional antibody responses to COVID-19 vaccination
Source: NPJ Vaccines. 2025 Oct 6;10:217. doi: 10.1038/s41541-025-01268-w (PMC12501030; doi:10.1038/s41541-025-01268-w)
Supplement: Supplementary file 1 — Supps Figures and Tables [file 41541_2025_1268_MOESM1_ESM.pdf]

Supplementary Table 1. Cohort demographics and clinical characteristics

	**2x BNT162b2**	**2x AZD1222**	**3x mRNA**	**2x AZD1222 + 1x mRNA**
**Control, *n***	30	29	20	21
Age, mean (range)	50 (27-67)	59 (29-82)	50 (28-66)	59 (42-82)
Female (%)	18 (60%)	16 (55%)	13 (65%)	12 (57%)
**STM, *n***	40	47	42	59
Age, (mean, range)	51 (26-76)	62 (39-88)	51 (23-84)	63 (38-78)
Female, *n* (%)	25 (63%)	23 (49%)	27 (64%)	33 (56%)
Metastatic disease, *n* (%)	22 (55%)	19 (40%)	24 (57%)	32 (54%)
Malignancy, *n* (%)				
Bone	0 (0%)	0 (0%)	1 (2%)	0 (0%)
Breast	7 (17.5%)	9 (19%)	8 (19%)	16 (27%)
Brain	9 (22.5%)	11 (23%)	8 (19%)	7 (12%)
Glioma	9 (100%)	9 (82%)	7 (87.5%)	6 (86%)
Meningioma	0 (0%)	2 (18%)	0 (0%)	1 (14%)
Other	0 (0%)	0 (0%)	1 (12.5%)	0 (0%)
Gastrointestinal	7 (17.5%)	4 (8.5%)	10 (24%)	10 (17%)
Colorectal	6 (86%)	2 (50%)	6 (60%)	3 (30%)
Gastric	0 (0%)	0 (0%)	0 (0%)	1 (10%)
GIST	0 (0%)	0 (0%)	1 (10%)	3 (30%)
Hepatic	0 (0%)	0 (0%)	1 (10%)	1 (10%)
Oesophageal	0 (0%)	0 (0%)	0 (0%)	1 (10%)
Pancreatic	1 (14%)	2 (50%)	2 (20%)	1 (10%)
Genitourinary	7 (17.5%)	6 (13%)	6 (14%)	6 (10%)
Prostate	1 (14%)	5 (83%)	1 (17%)	4 (67%)
Testicular	1 (14%)	0 (0%)	1 (17%)	0 (0%)
Renal	4 (47%)	1 (17%)	3 (50%)	2 (33%)
Urothelial	1 (14%)	0 (0%)	1 (17%)	0 (0%)
Gynaecological	4 (10%)	4 (8.5%)	5 (12%)	8 (14%)
Cervical	1 (25%)	0 (0%)	1 (20%)	0 (0%)
Ovarian	3 (75%)	1 (25%)	4 (80%)	3 (37.5%)
Uterine	0 (0%)	3 (75%)	0 (0%)	5 (62.5%)
Head and Neck	2 (5%)	3 (6%)	1 (2%)	2 (3%)
Nasopharyngeal	2 (100%)	0 (0%)	1 (100%)	0 (0%)
Oropharyngeal	0 (0%)	3 (100%)	0 (0%)	2 (100%)
Lung	4 (10%)	2 (4%)	3 (7%)	1 (2%)
NSCLC	2 (50%)	2 (100%)	1 (33%)	1 (100%)
Mesothelioma	2 (50%)	0 (0%)	2 (67%)	0 (0%)
Skin	0 (0%)	8 (17%)	0 (0%)	7 (12%)
Melanoma	0 (0%)	4 (50%)	0 (0%)	4 (57%)
Other	0 (0%)	4 (50%)	0 (0%)	3 (43%)
Treatment, *n* (%)				
Chemotherapy	20 (50%)	16 (34%)	23 (55%)	21 (36%)
Immunotherapy	7 (17.5%)	11 (23%)	4 (9.5%)	9 (15%)
Targeted therapy	5 (12.5%)	6 (13%)	11 (26%)	12 (31%)
Radiotherapy	1 (2.5%)	1 (2%)	0 (0%)	0 (0%)
Observation following previous chemotherapy	5 (12.5%)	7 (15%)	3 (7%)	8 (14%)
Observation following previous immunotherapy	2 (5%)	1 (2%)	1 (2%)	1 (2%)
Observation following previous radiotherapy	0 (0%)	3 (6%)	0 (0%)	1 (2%)
Best supportive care	0 (0%)	2 (4%)	0 (0%)	0 (0%)

Chemotherapy: any patient who received chemotherapy; Immunotherapy: any patient who received immunotherapy, but not chemotherapy; Targeted therapy: any patient who received targeted therapy, but did not receive chemotherapy nor immunotherapy; Radiotherapy: any patient who received radiotherapy, but did not receive chemotherapy, immunotherapy, nor targeted therapy. Abbreviations: STM, solid tumor malignancy; GIST, gastrointestinal stromal tumor; NSCLC, non-small cell lung cancer.

Supplementary Table 2. Details of biotinylated mouse anti-human anti-IgG antibody detectors and soluble FcγR and C1q reagents used in bead-based multiplex assays

Detector	Clone	Manufacturer	Catalogue number	Plasma dilution
Total IgA, biotin	MT20	MabTech	3860-6-250	1:250
Total IgG, biotin	MT78/145	MabTech	3850-6-250	1:3200
IgG1, biotin	HP1218	MabTech	3851-14-250	1:1600
IgG2, biotin	HP6200	MabTech	3852-6-250	1:250
IgG3, biotin	MTG34	MabTech	3853-6-250	1:250
IgG4, biotin	MTG42	MabTech	3854-6-250	1:1600
FcγR2a-H131	N/A	Produced in-house	PMID: 27385782	1:600
FcγR2a-R131	N/A	Produced in-house	PMID: 27385782	1:600
FcγR2b	N/A	Produced in-house	N/A	1:600
FcγR3a-V158	N/A	Produced in-house	PMID: 27385782	1:600
FcγR2a-F158	N/A	Produced in-house	PMID: 27385782	1:600
C1q	N/A	Sigma-Aldrich	C1740-.5MG	1:600

Dilutions indicate the final dilution of plasma (following addition of bead cocktail) used in multiplex assays.

Supplementary Table 3. Details of antibodies used for detection of memory B cells

Detector	Clone	Fluorochrome	Manufacturer	Antibody dilution
IgD	Goat polyclonal	FITC	SouthernBiotech	1:100
CD20	2H7	APC-C7/NIR	BioLegend	1:100
CD14	M5E2	BV510	BioLegend	1:200
CD3	OKT3	BV510	BioLegend	1:400
CD8a	RPA-T8	BV510	BioLegend	1:1000
CD16	3G8	BV510	BioLegend	1:300
CD10	HI10a	BV510	BioLegend	1:500
CD27	O323	BV605	BioLegend	1:100
CD11c	B-ly6	BV650	BD Biosciences	1:50
IgG	G18-145	BV786	BD Biosciences	1:50
CD19	J3-119	PECF594	Beckman Coulter	1:100
CD71	CY1G4	PE-Cy7	BioLegend	1:50
FcRL5	509F6	BUV395	BD Biosciences	1:50
CD21	B-ly4	BUV737	BD Biosciences	1:100

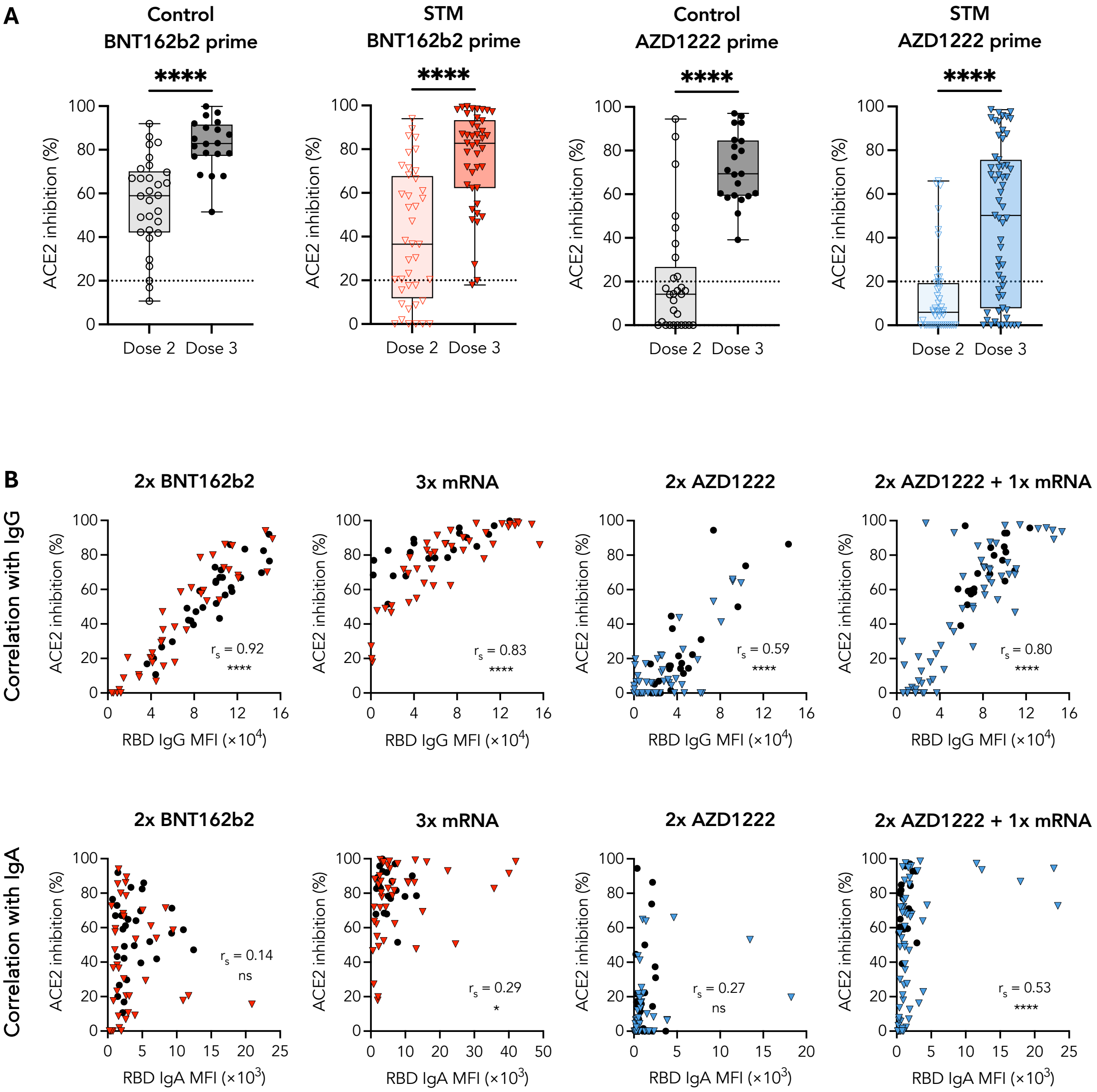

Supplementary Fig. 1. RBD-specific IgG underpins robust neutralizing capacity following mRNA-based booster vaccination. A) Neutralizing capacity of STM and control vaccinees one month post second (BNT162b2 or AZD1222) and one month post third (mRNA) dose of the indicated vaccine regimen. **B)** Spearman correlations of IgG or IgA with ACE2 inhibition one month post indicated vaccine regimen. Mann-Whitney *U*-tests performed between STM and control vaccinees within each vaccinee regimen. $P < 0.0001$ (****); $P < 0.05$ (*); non-significant (ns). MFI: median fluorescence intensity.

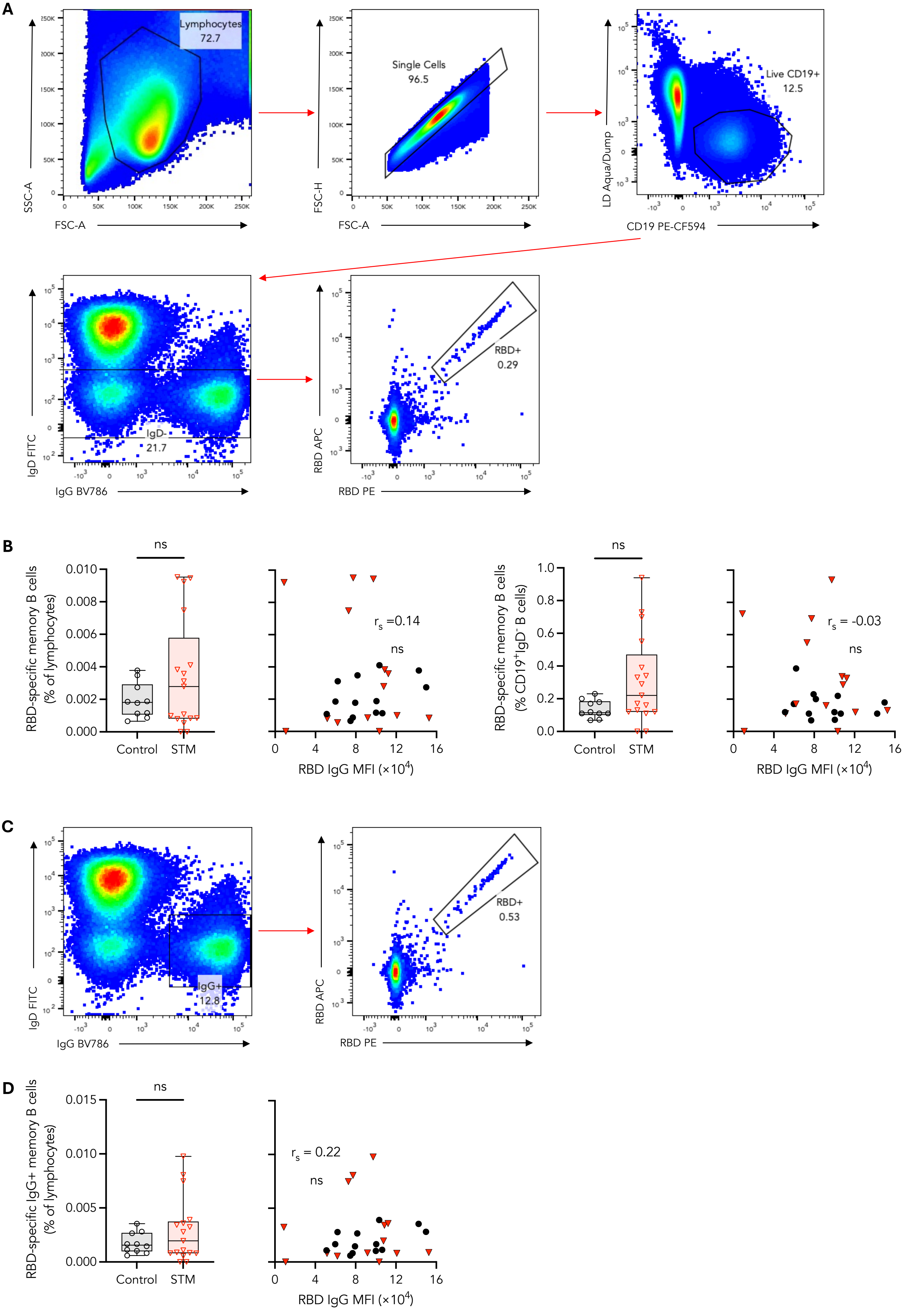
A
Lymphocytes
72.7
SSC-A
FSC-A
Single Cells
96.5
FSC-H
FSC-A
Live CD19+
12.5
LD Aqua/Dump
CD19 PE-CF594
IgD-
21.7
IgD FITC
IgG BV786
RBD+
0.29
RBD APC
RBD PE
B
ns
RBD-specific memory B cells
(% of lymphocytes)
Control
STM
r_s =0.14
ns
RBD IgG MFI (×10^4)
ns
RBD-specific memory B cells
(% CD19+IgD- B cells)
Control
STM
r_s = -0.03
ns
RBD IgG MFI (×10^4)
C
IgG+
12.8
IgD FITC
IgG BV786
RBD+
0.53
RBD APC
RBD PE
D
ns
RBD-specific IgG+ memory B cells
(% of lymphocytes)
Control
STM
r_s = 0.22
ns
RBD IgG MFI (×10^4)

Supplementary Fig. 2. Robust RBD-specific memory B cells in STM patients. A) Gating strategy for identification of RBD-specific switched memory B cells. **B)** RBD-specific switched memory B cells one month post second BNT162b2 dose as a frequency of IgD^- B cells or total lymphocytes. Spearman correlations of RBD-specific switched memory B cells and RBD-specific IgG one month post second BNT162b2 dose. **C)** Gating strategy for identification of RBD-specific IgG^+ memory B cells. **D)** RBD-specific IgG^+ memory B cells one month post second BNT162b2 dose as a frequency of total lymphocytes. Spearman correlations of RBD-specific IgG^+ memory B cells and RBD-specific IgG one month post second BNT162b2 dose. Mann-Whitney *U*-tests performed between STM and control vaccinees within each vaccinee regimen. non-significant (ns). MFI: median fluorescence intensity.

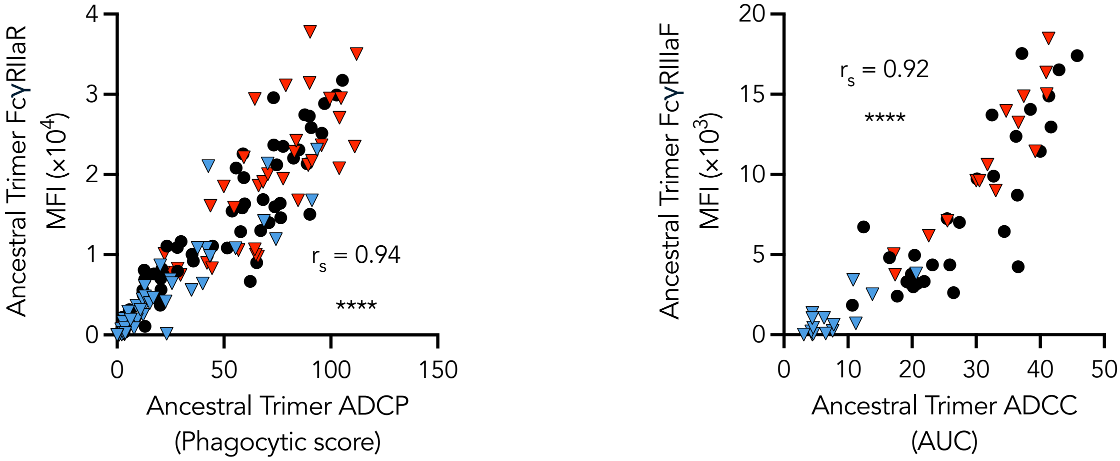

Supplementary Fig. 3. ADCP and ADCC correlate with engagement of lower-affinity FcγRIIaR and FcγRIIIaF. Spearman correlation of ADCP with FcγRIIaR binding. Spearman correlation of ADCC with FcγRIIIaF binding. $P < 0.0001$ (****). MFI: median fluorescence intensity. AUC: area under curve.

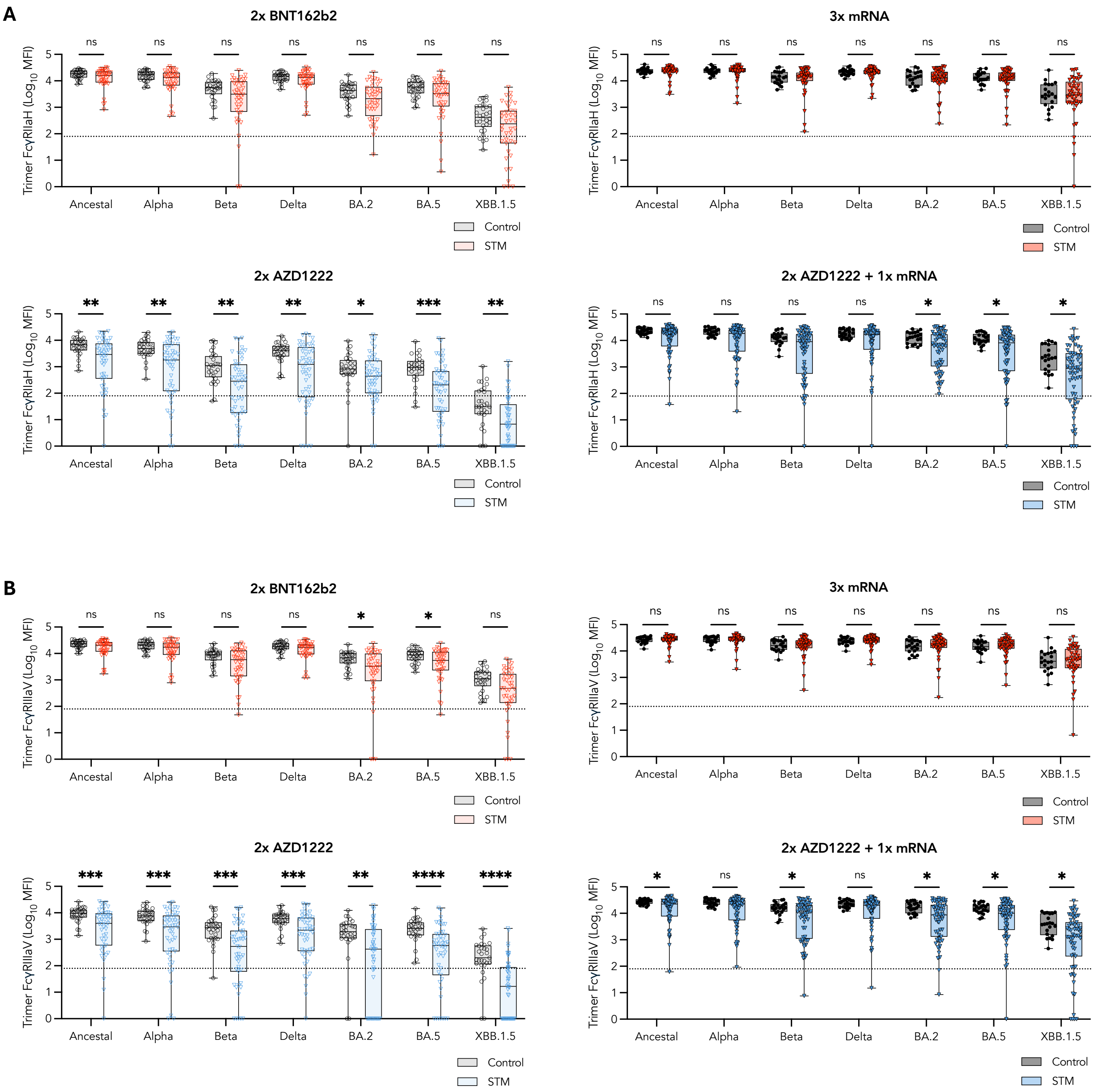

Supplementary Fig. 4. Robust variant cross-reactive FcγR binding in STM patients. SARS-CoV-2 ancestral S2-, trimer-, S1-, and RBD- specific engagement of FcγRIIaH and FcγRIIIaV one month post the indicated vaccine regimen in **A)** BNT162b2- or **B)** AZD1222- primed vaccinees. Mann-Whitney *U*-tests performed between STM and control vaccinees within each vaccinee regimen. $P < 0.0001$ (****); $P < 0.001$ (***); $P < 0.01$ (**); $P < 0.05$ (*); non-significant (ns). MFI: median fluorescence intensity.

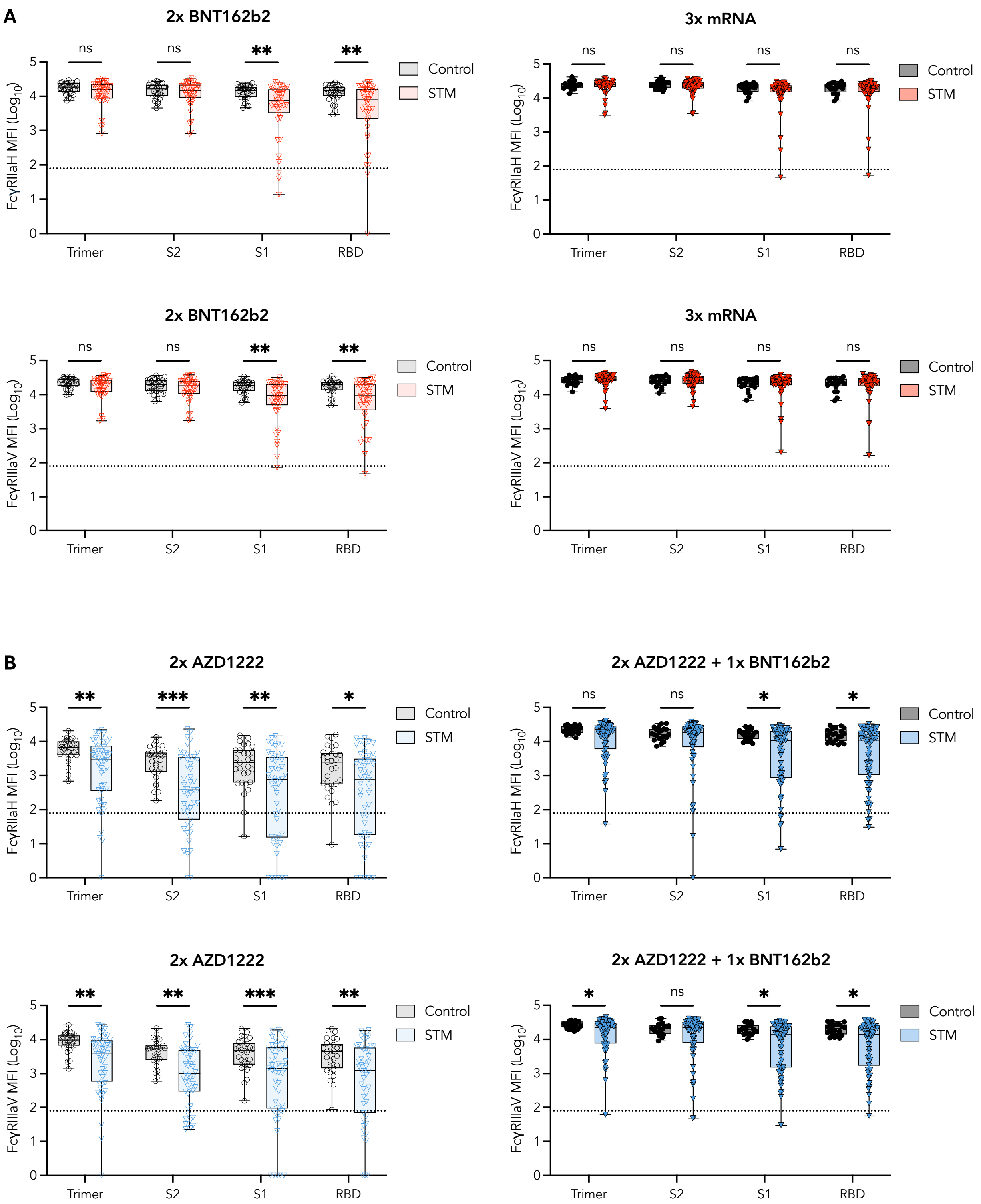

Supplementary Fig. 5. Robust variant cross-reactive FcγR binding in STM patients. SARS-CoV-2 variant trimer-specific engagement of **A)** FcγRIIaH and **B)** FcγRIIIaV one month post the indicated vaccine regimen. Mann-Whitney *U*-tests performed between STM and control vaccinees within each vaccinee regimen. $P < 0.0001$ (****); $P < 0.001$ (***); $P < 0.01$ (**); $P < 0.05$ (*); non-significant (ns). MFI: median fluorescence intensity.

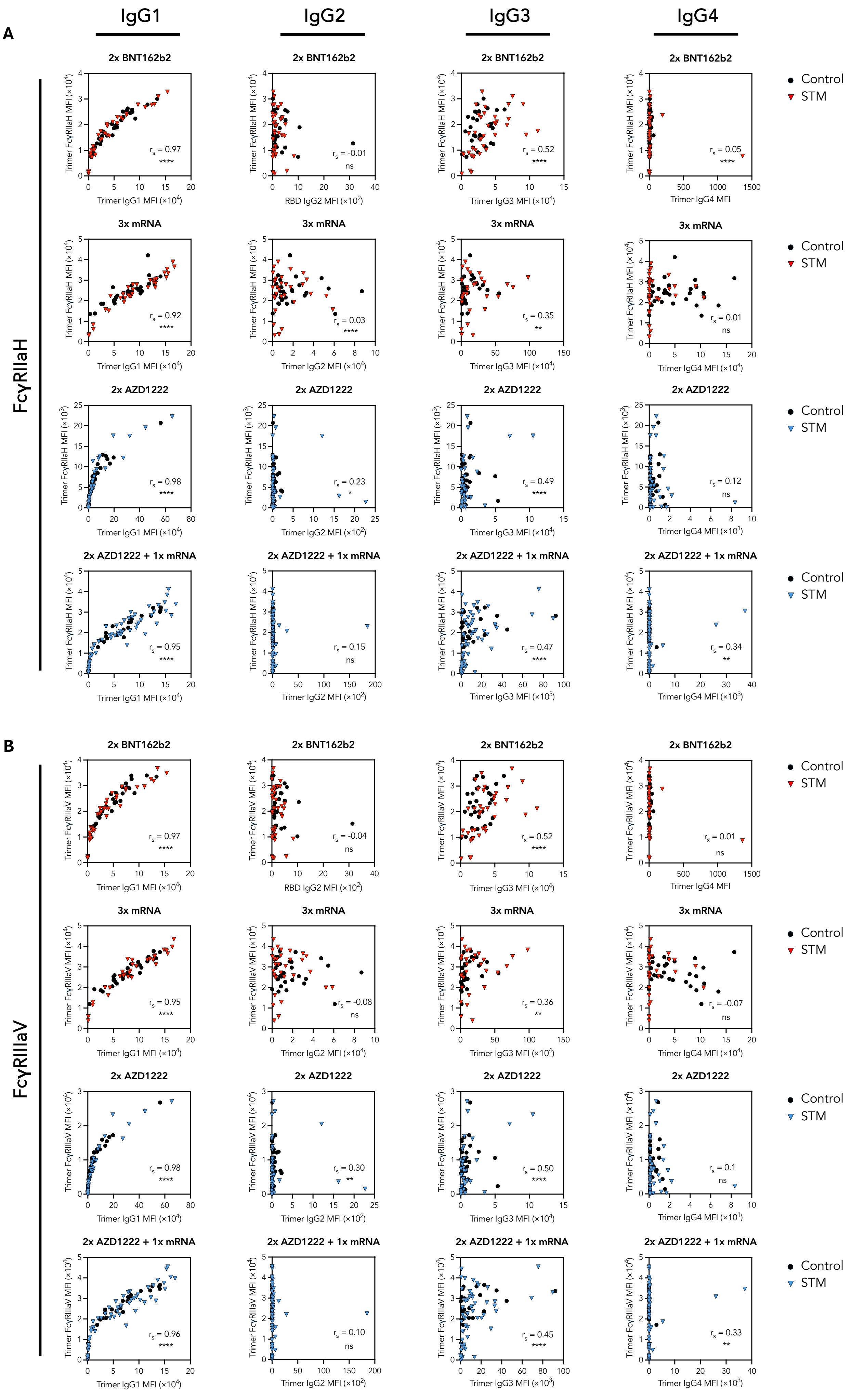

A
IgG1
IgG2
IgG3
IgG4
FcγRIIaH
2x BNT162b2
Trimer FcγRIIaH MFI (×10^4)
Trimer IgG1 MFI (×10^4)
r_s = 0.97
****
RBD IgG2 MFI (×10^2)
r_s = -0.01
ns
Trimer IgG3 MFI (×10^4)
r_s = 0.52
****
Trimer IgG4 MFI
r_s = 0.05
****
Control
STM
3x mRNA
Trimer IgG1 MFI (×10^4)
r_s = 0.92
****
Trimer IgG2 MFI (×10^4)
r_s = 0.03
****
Trimer IgG3 MFI (×10^4)
r_s = 0.35
**
Trimer IgG4 MFI (×10^4)
r_s = 0.01
ns
Control
STM
2x AZD1222
Trimer FcγRIIaH MFI (×10^3)
Trimer IgG1 MFI (×10^4)
r_s = 0.98
****
Trimer IgG2 MFI (×10^2)
r_s = 0.23
*
Trimer IgG3 MFI (×10^4)
r_s = 0.49
****
Trimer IgG4 MFI (×10^1)
r_s = 0.12
ns
Control
STM
2x AZD1222 + 1x mRNA
Trimer FcγRIIaH MFI (×10^4)
Trimer IgG1 MFI (×10^4)
r_s = 0.95
****
Trimer IgG2 MFI (×10^2)
r_s = 0.15
ns
Trimer IgG3 MFI (×10^3)
r_s = 0.47
****
Trimer IgG4 MFI (×10^3)
r_s = 0.34
**
Control
STM
B
FcγRIIIaV
2x BNT162b2
Trimer FcγRIIIaV MFI (×10^4)
Trimer IgG1 MFI (×10^4)
r_s = 0.97
****
RBD IgG2 MFI (×10^2)
r_s = -0.04
ns
Trimer IgG3 MFI (×10^4)
r_s = 0.52
****
Trimer IgG4 MFI
r_s = 0.01
ns
Control
STM
3x mRNA
Trimer IgG1 MFI (×10^4)
r_s = 0.95
****
Trimer IgG2 MFI (×10^4)
r_s = -0.08
ns
Trimer IgG3 MFI (×10^4)
r_s = 0.36
**
Trimer IgG4 MFI (×10^4)
r_s = -0.07
ns
Control
STM
2x AZD1222
Trimer IgG1 MFI (×10^4)
r_s = 0.98
****
Trimer IgG2 MFI (×10^2)
r_s = 0.30
**
Trimer IgG3 MFI (×10^4)
r_s = 0.50
****
Trimer IgG4 MFI (×10^1)
r_s = 0.1
ns
Control
STM
2x AZD1222 + 1x mRNA
Trimer IgG1 MFI (×10^4)
r_s = 0.96
****
Trimer IgG2 MFI (×10^2)
r_s = 0.10
ns
Trimer IgG3 MFI (×10^3)
r_s = 0.45
****
Trimer IgG4 MFI (×10^3)
r_s = 0.33
**
Control
STM

Supplementary Fig. 6. Trimer-specific IgG1 responses underpin trends in FcγR binding. Spearman correlations of SARS-CoV-2 trimer-specific **A)** FcγRIIaH and **B)** FcγRIIIaV with IgG1-4 one month post the indicated vaccine regimen. $P < 0.0001$ (****); $P < 0.01$ (**); $P < 0.05$ (*); non-significant (ns). MFI: median fluorescence intensity.

BNT162b2 prime

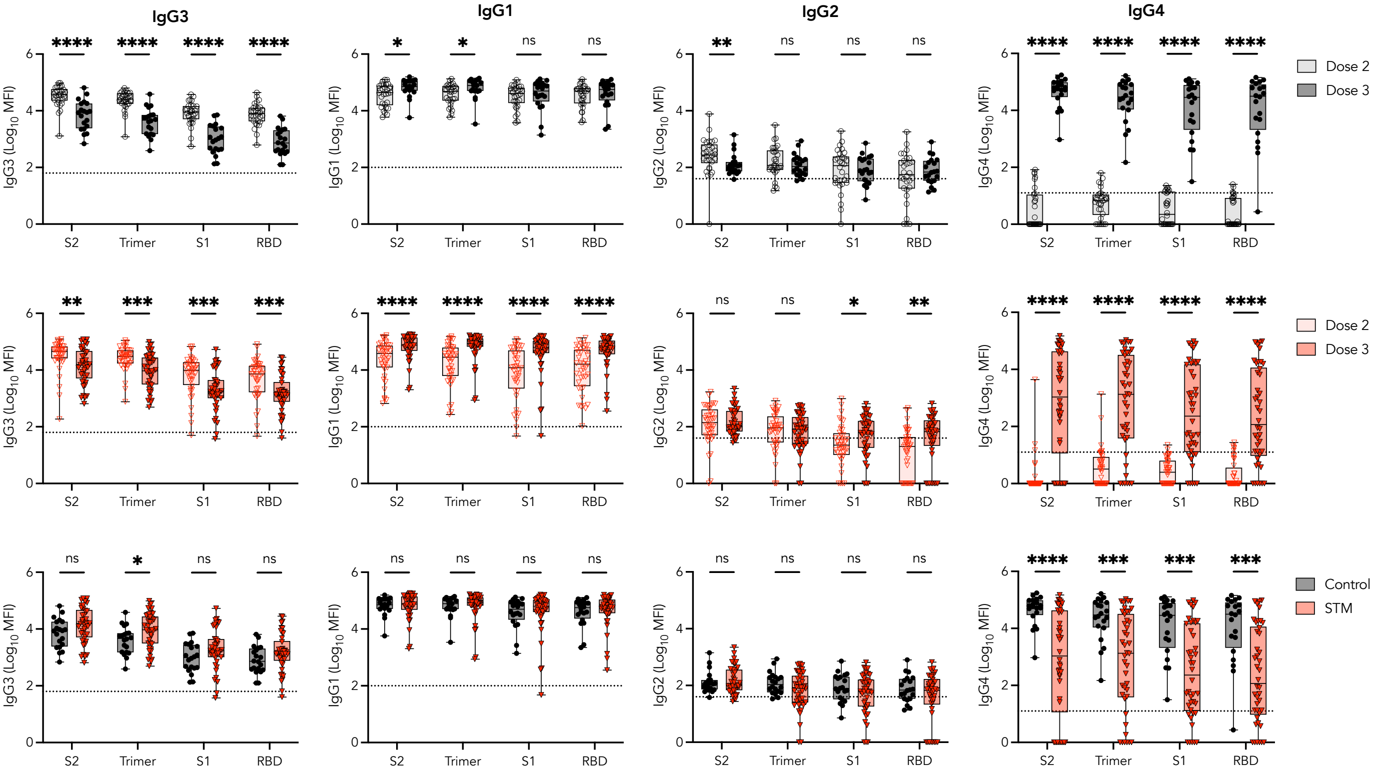

AZD1222 prime

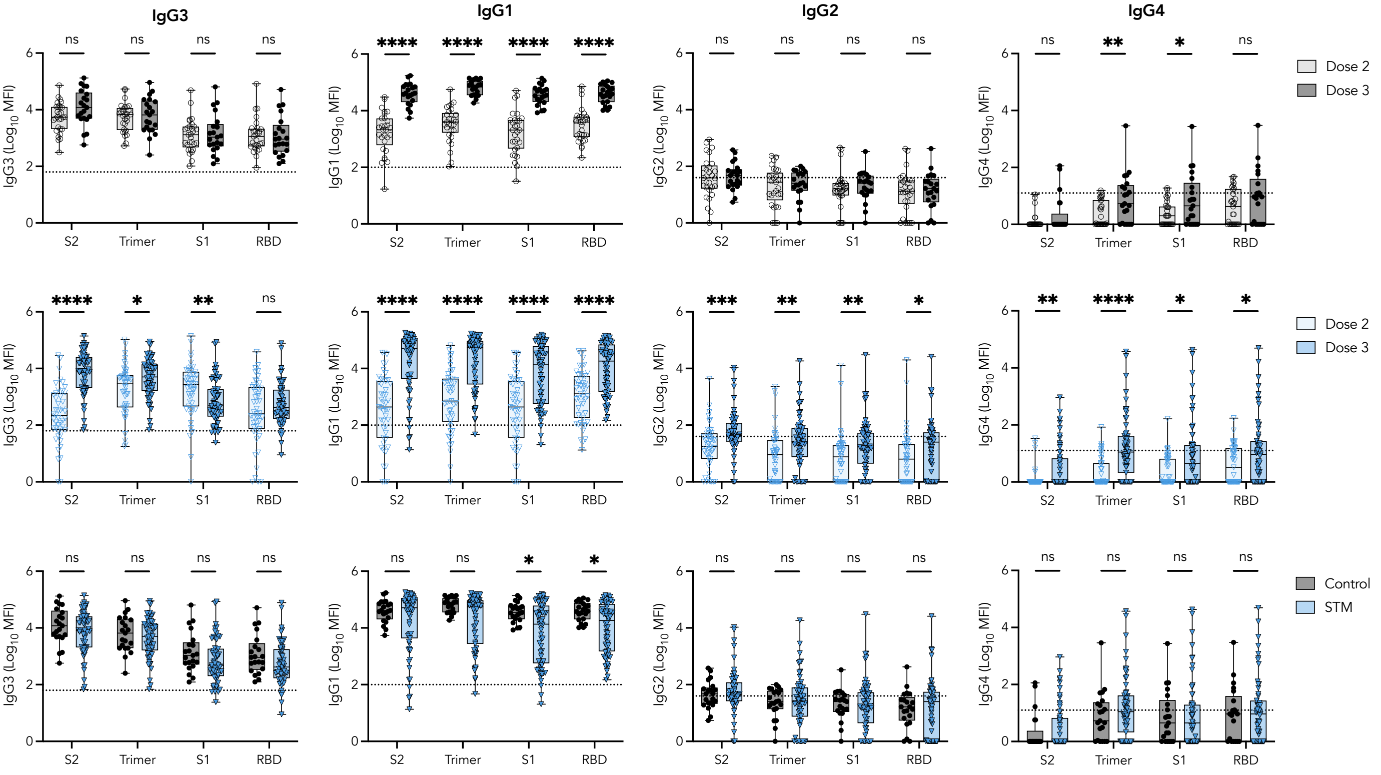

Supplementary Fig. 7. IgG subclass switching in BNT162b2- and AZD1222- primed patients and controls following a third mRNA vaccination. SARS-CoV-2 S2-, trimer-, S1-, and RBD-specific IgG1-IgG4 responses one month post dose 2 of the indicated priming regimen and one month post mRNA-based third vaccination. Mann-Whitney *U*-tests performed between STM and control vaccinees or between dose 2 and dose 3 responses within each vaccinee regimen. $P <$ 0.0001 (****); $P < 0.001$ (***); $P < 0.01$ (**); $P < 0.05$ (*); non-significant (ns). MFI: median fluorescence intensity.

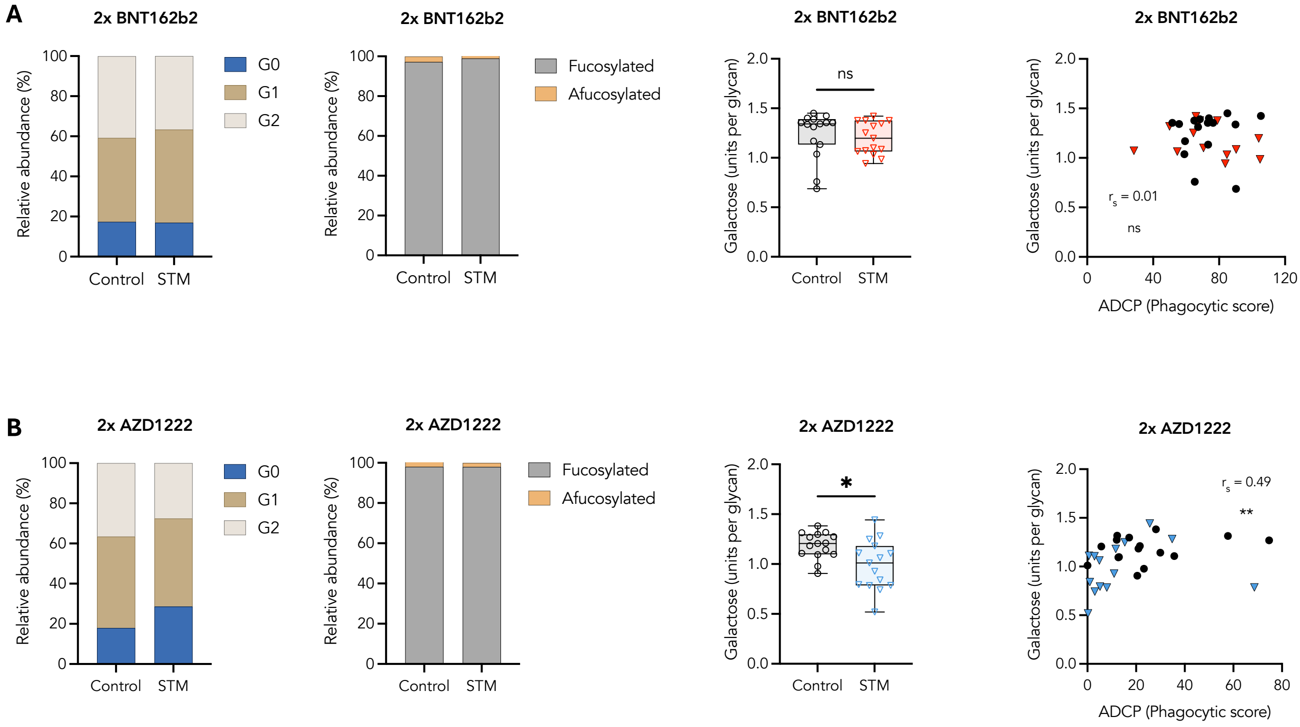

Supplementary Fig. 8. Modulation of antigen-specific IgG glycosylation profiles and association with Fc effector function following COVID-19 vaccination. RBD-specific IgG galactosylation and fucosylation and Spearman correlations between galactosylation and ADCP one month post two **A)** BNT162b2 doses (n = 15 control; n = 15 STM) or **B)** AZD1222 doses (n = 15 control; n = 15 STM). Mann-Whitney U-tests performed between STM and control vaccinees within each vaccinee regimen. $P < 0.01$ (**); $P < 0.05$ (*); non-significant (ns).

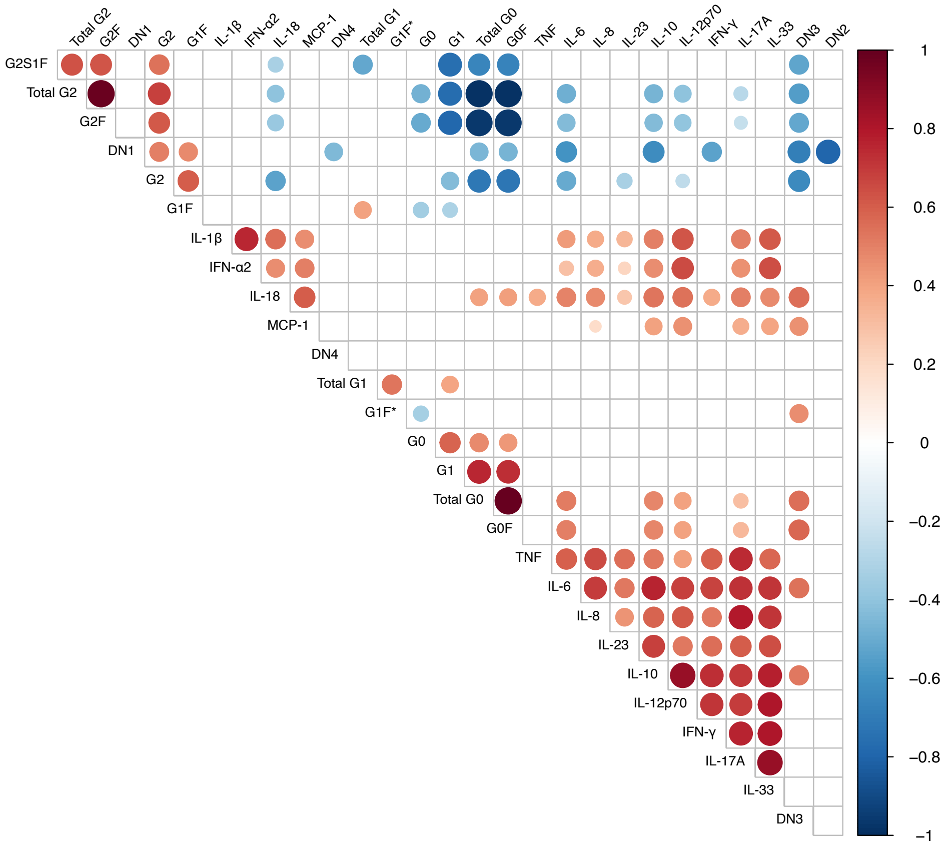

Supplementary Fig. 9. Correlations between inflammatory biomarkers. Correlation matrix showing Spearman correlations between inflammatory biomarkers in control and STM vaccinees one month post two BNT162b2 doses. Statistical significance was determined with an unpaired, two-sided *t*-test with Benjamini–Hochberg adjustment. Size of each circle indicates magnitude of the correlation coefficient. Only significant correlations are shown; non-significant correlations are indicated as white boxes.

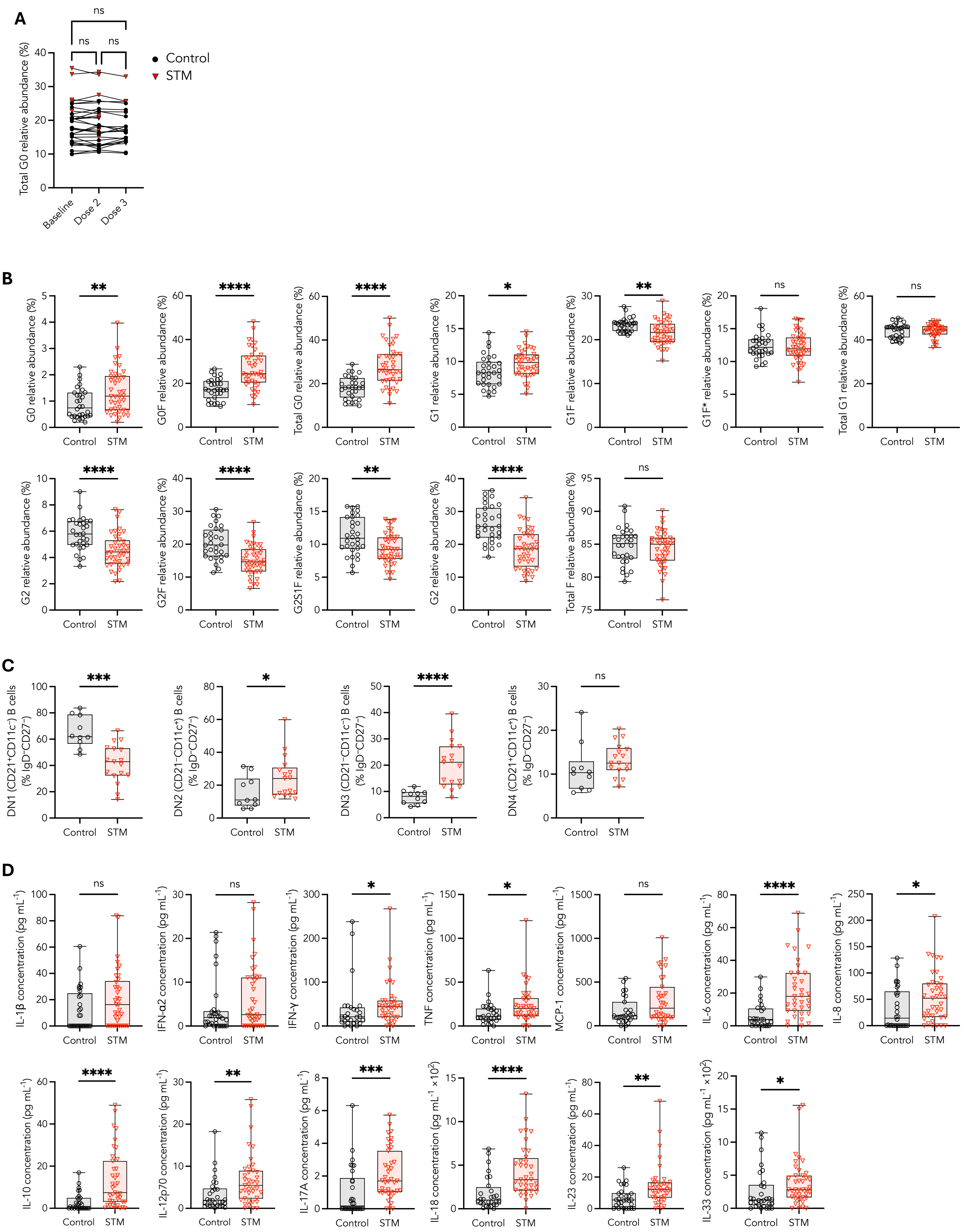
A
B
C
D
Control
STM
ns
Total G0 relative abundance (%)
Baseline
Dose 2
Dose 3
G0 relative abundance (%)
G0F relative abundance (%)
Total G0 relative abundance (%)
G1 relative abundance (%)
G1F relative abundance (%)
G1F* relative abundance (%)
Total G1 relative abundance (%)
G2 relative abundance (%)
G2F relative abundance (%)
G2S1F relative abundance (%)
G2 relative abundance (%)
Total F relative abundance (%)
DN1 (CD21⁺CD11c⁻) B cells (% IgD⁻CD27⁻)
DN2 (CD21⁻CD11c⁺) B cells (% IgD⁻CD27⁻)
DN3 (CD21⁻CD11c⁻) B cells (% IgD⁻CD27⁻)
DN4 (CD21⁺CD11c⁺) B cells (% IgD⁻CD27⁻)
IL-1β concentration (pg mL⁻¹)
IFN-α2 concentration (pg mL⁻¹)
IFN-γ concentration (pg mL⁻¹)
TNF concentration (pg mL⁻¹)
MCP-1 concentration (pg mL⁻¹)
IL-6 concentration (pg mL⁻¹)
IL-8 concentration (pg mL⁻¹)
IL-10 concentration (pg mL⁻¹)
IL-12p70 concentration (pg mL⁻¹)
IL-17A concentration (pg mL⁻¹)
IL-18 concentration (pg mL⁻¹ ×10²)
IL-23 concentration (pg mL⁻¹)
IL-33 concentration (pg mL⁻¹ ×10²)

Supplementary Fig. 10. Inflammatory biomarkers in STM patients and control vaccinees. **A)** Abundance of total agalactosylated (G0) IgG glycoforms over the course of a three-dose BNT162b2 vaccination regimen. Baseline: pre-vaccination; Dose 2: one month post second dose; Dose 3: one month post third dose. **B)** Relative abundance of IgG glycoforms, **C)** relative frequencies of DN memory B cell subsets, and **D)** concentration of 13 cytokines one month post second BNT162b2 vaccination. Kruskal-Wallis test performed to assess changes in IgG glycosylation across timepoints. Mann-Whitney *U*-tests performed between STM and control vaccinees. $P < 0.0001$ (****); $P < 0.001$ (***); $P < 0.01$ (**); $P < 0.05$ (*); non-significant (ns). MFI: median fluorescence intensity.

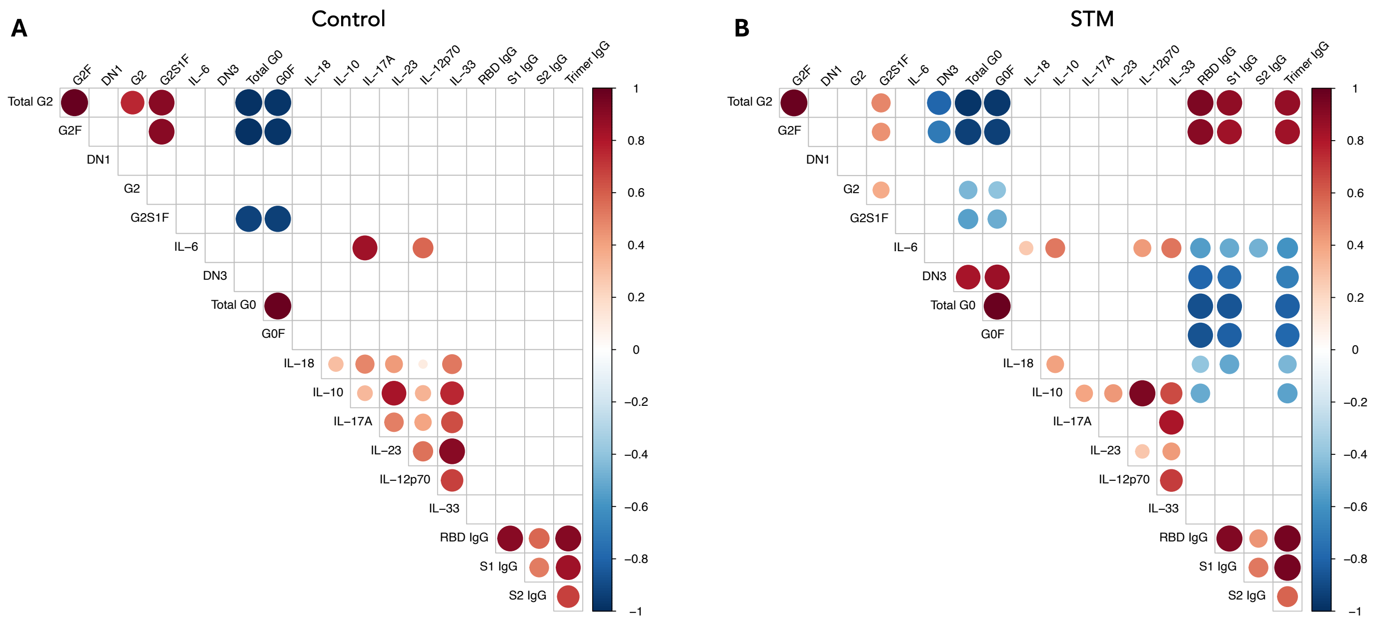

Supplementary Fig. 11. Correlations between inflammatory biomarkers and SARS-CoV-2-specific IgG. A-B) Correlation matrices showing the Spearman correlations between SARS-CoV-2-specific IgG responses and differentially abundant inflammatory biomarkers for **A)** control and **B)** STM vaccinees one month post two BNT162b2 doses. Statistical significance was determined with an unpaired, two-sided *t*-test with Benjamini–Hochberg adjustment. Size of each circle indicates magnitude of the correlation coefficient. Only significant correlations are shown; non-significant correlations are indicated as white boxes.

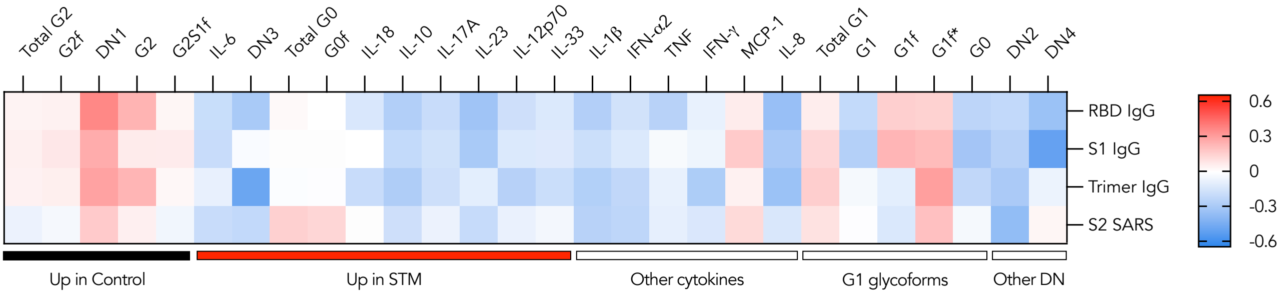

Supplementary Fig. 12. Correlations between inflammatory biomarkers and SARS-CoV-2-specific IgG one month post third mRNA vaccination. Spearman correlations between differentially abundant inflammatory biomarkers and SARS-CoV-2-specific IgG responses for control and STM vaccinees one month post third mRNA vaccination. Statistical significance adjusted for multiple comparisons via Holm-Šídák method. No significant correlations were identified.

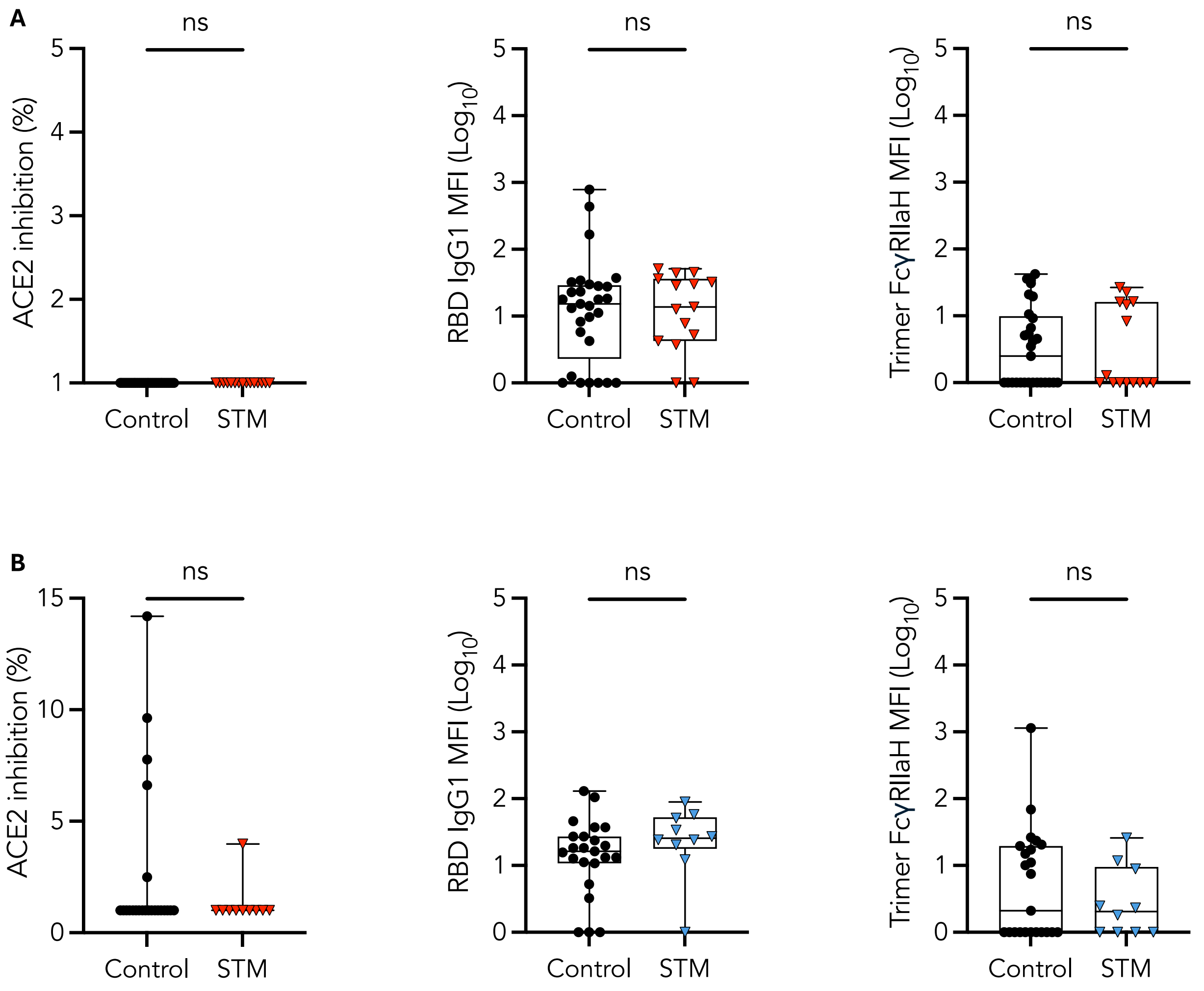

Supplementary Fig. 13. Comparable pre-vaccination baseline SARS-CoV-2 binding and functional antibody reactivity between STM and control vaccinees. Ancestral SARS-CoV-2 neutralizing capacity, RBD-specific IgG1, and trimer-specific FcγRIIaH responses in STM and control vaccinees prior to primary **A)** BNT162b2 or **B)** AZD1222 vaccination. Unpaired, nonparametric rank order comparisons between STM and control vaccinees within each vaccinee regimen determined via Mann-Whitney *U*-tests. non-significant (ns). MFI: median fluorescence intensity.

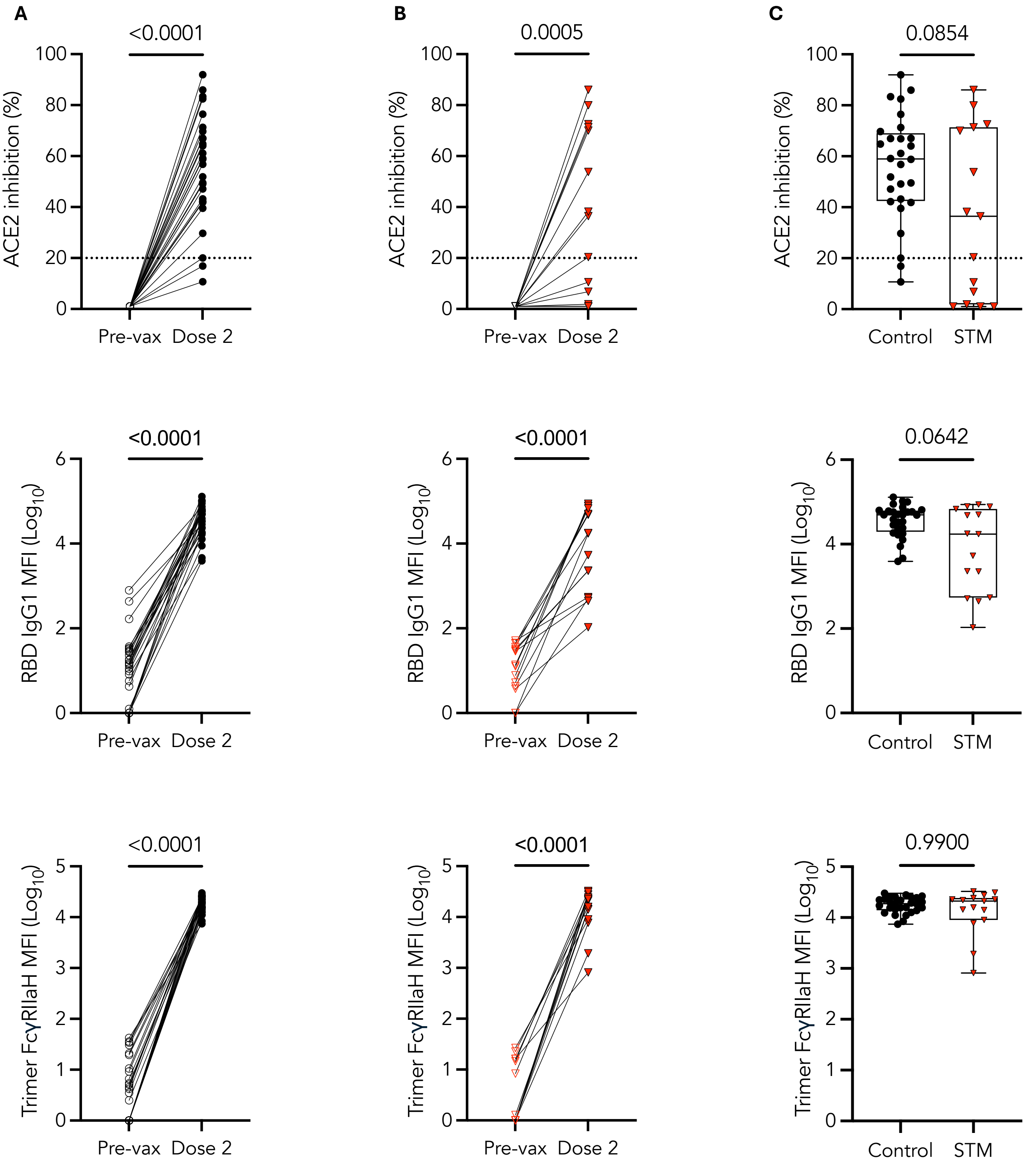

Supplementary Fig. 14. Vaccinees for whom paired samples collected at baseline and one month post dose two BNT162b2 were available. Responses compared between baseline and dose two for **A)** controls and **B)** STM vaccinees. Paired comparisons between pre-vaccination and post dose two responses determined nonparametric Wilcoxon matched-pairs signed rank test. **C)** Comparison of responses between STM and control vaccinees to ensure consist trends between the larger cross-sectional cohort and subset for whom longitudinal samples were available. Unpaired, nonparametric rank order comparisons between STM and control vaccinees within each vaccinee regimen determined via Mann-Whitney *U*-tests. *P*-values denoted numerically above horizonal bars on each graph. non-significant (ns). MFI: median fluorescence intensity.

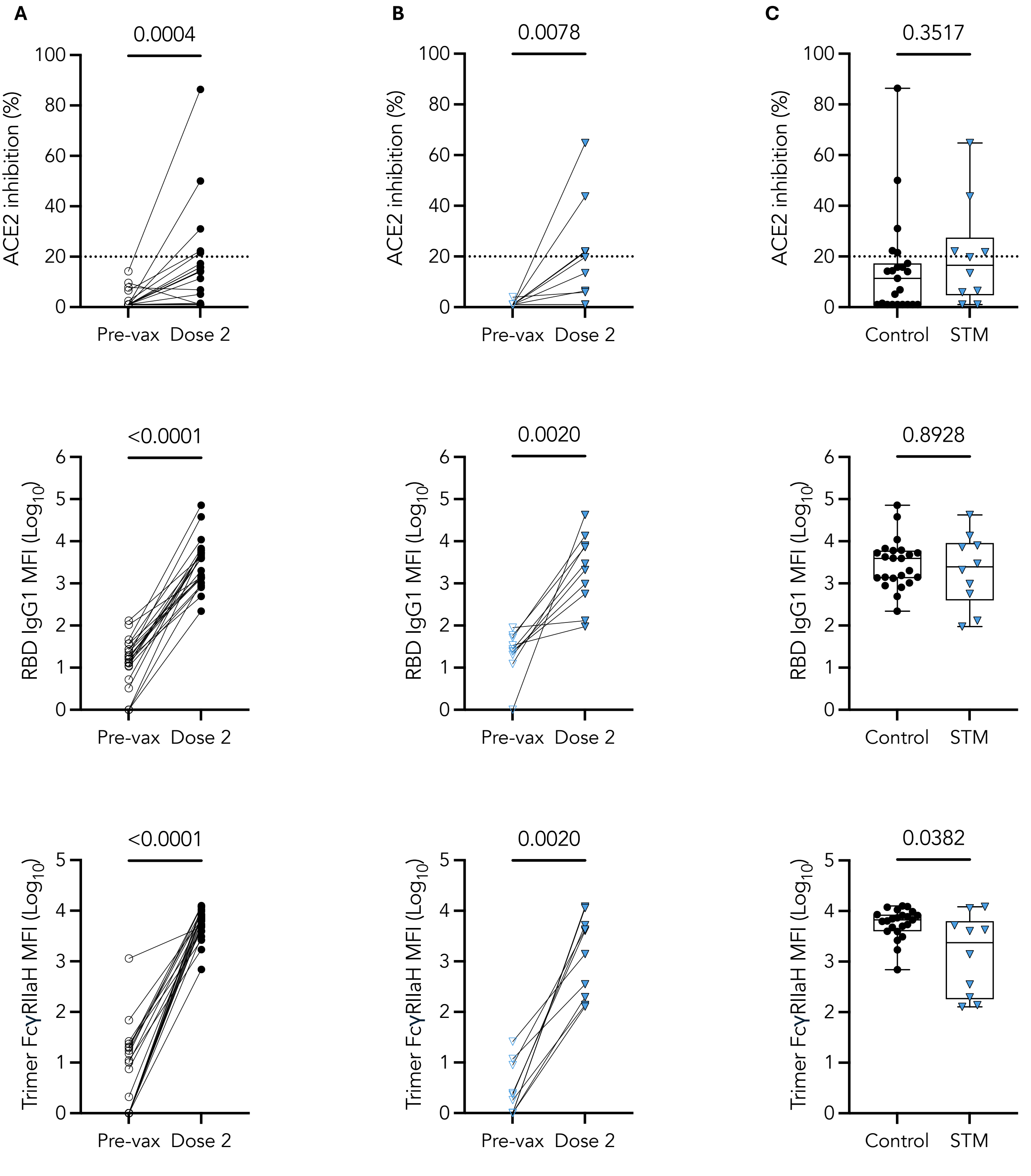

Supplementary Fig. 15. Vaccinees for whom paired samples collected at baseline and one month post dose two AZD1222 were available. Responses compared between baseline and dose two for **A)** controls and **B)** STM vaccinees. Paired comparisons between pre-vaccination and post dose two responses determined nonparametric Wilcoxon matched-pairs signed rank test. **C)** Comparison of responses between STM and control vaccinees to ensure consist trends between the larger cross-sectional cohort and subset for whom longitudinal samples were available. Unpaired, nonparametric rank order comparisons between STM and control vaccinees within each vaccinee regimen determined via Mann-Whitney *U*-tests. *P*-values denoted numerically above horizonal bars on each graph. non-significant (ns). MFI: median fluorescence intensity.
